# Small Bowel Obstruction Secondary to Migration of a Fractured Esophageal Stent

**DOI:** 10.7759/cureus.30802

**Published:** 2022-10-28

**Authors:** Yousif Abdallah Adam, Sean-Tee J.M. Lim, Fionnuala Redmond, Eanna J. Ryan, Sean Johnston

**Affiliations:** 1 Vascular Surgery, University Hospital Waterford, Waterford, IRL; 2 Radiology, Tallaght University Hospital, Dublin, IRL; 3 General Surgery, Midland Regional Hospital Tullamore, Tullamore, IRL; 4 General and Colorectal Surgery, Tallaght University Hospital, Dublin, IRL

**Keywords:** boerhaave's syndrome, conservative management, endoscopic approach, intestinal obstruction, esophageal stent

## Abstract

Esophageal stent placement is commonly indicated for the management of inoperable esophageal malignancies, benign strictures, and esophageal perforations including Boerhaave’s syndrome. We present a case of a 74-year-old female, who presented with small bowel obstruction secondary to a migrated esophageal stent, which was placed 20 weeks previously for Boerhaave’s syndrome. She was surgically managed with laparotomy and retrieval of the fractured stent with local resection of the small bowel, followed by primary anastomosis.

## Introduction

Endoscopic esophageal stent placement has a wide range of utility, ranging from use for benign strictures to esophageal perforations, to fistulas and for palliative therapy of esophageal cancer [1,2]. There has been an increasing use of stenting for the management of esophageal perforation over operative repair due to its less invasive nature [3]. Endoluminal stent placement provides rapid closure of the perforation, eliminating soilage of the mediastinum, pleura, and peritoneum while avoiding the trauma of an urgent thoracotomy [4]. While stent placement is often a safe and effective treatment, a number of complications have been recognized, including stent migration, tracheoesophageal fistula, hemorrhage, obstruction, and gastrointestinal perforation [5]. We report a case of a 74-year-old female who presented with small bowel obstruction secondary to a fractured esophageal stent.

## Case presentation

A 74-year-old female presented to our medical center with a two-week history of lower abdominal pain and nausea. On examination, there was tenderness in the lower abdomen with guarding. Her past medical history included total abdominal hysterectomy with bilateral salpingo-oophorectomy, vaginal prolapse, bilateral mastectomy for breast cancer, and thyroidectomy.

**Figure 1:**
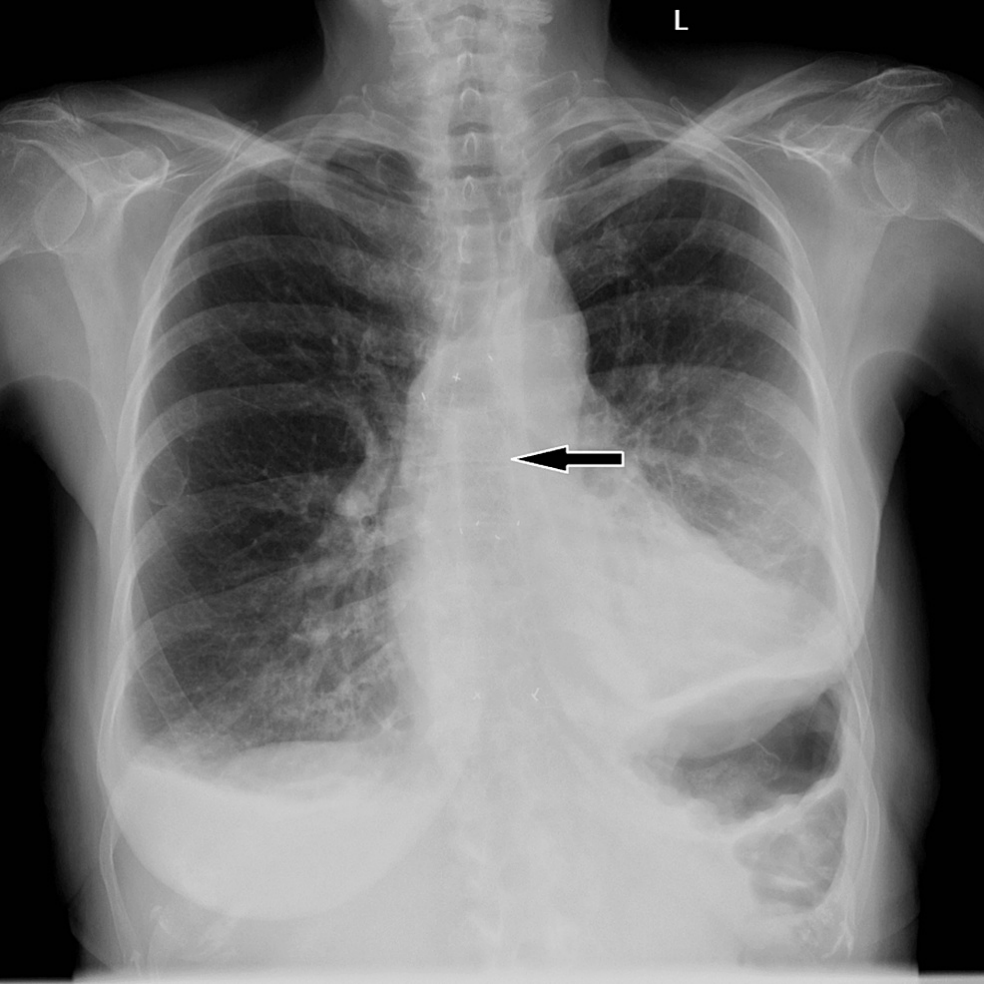
Chest radiograph after the initial esophageal stent placement (black arrow).

She had a background history of Boerhaave’s syndrome with a distal ruptured esophagus 20 weeks prior. At that time, she was thought to have first presented with partial left pneumothorax and pneumomediastinum, which was managed with chest drain insertion. A delayed diagnosis of a distal ruptured esophagus lead her to being treated conservatively at that time with an endoscopically inserted self-expanding covered stent (full covered type) 18 × 150 mm in diameter at the distal esophagus. Correct placement of the stent in the distal esophagus is demonstrated on the chest radiograph (Figure 1).

She had a trial of removal of the stent five months after placement, although the stent was located in the esophagus, but the trial was unsuccessful at endoscopy. She was asymptomatic with the stent, and it was therefore left in situ.

**Figure 2:**
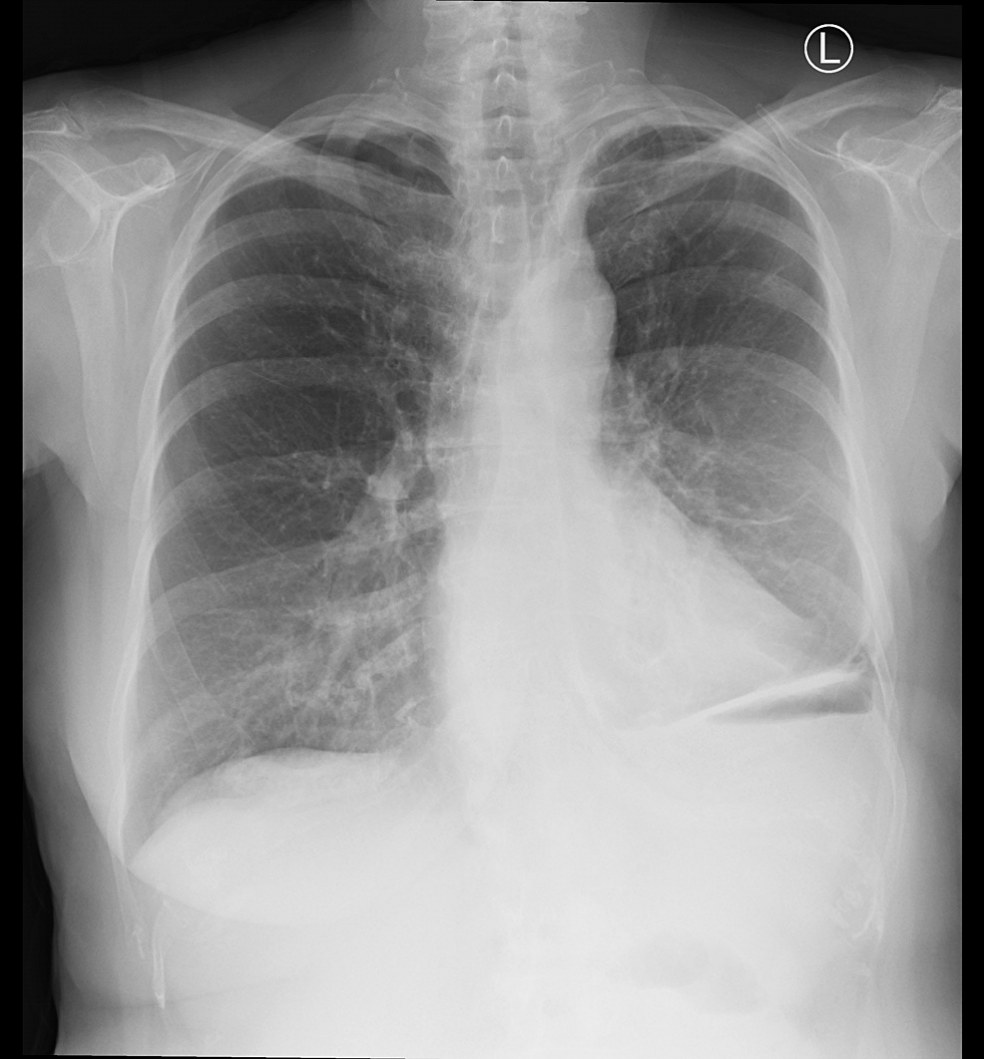
Chest radiograph on admission demonstrating the absence of the stent in the mediastinum.

On her admission, laboratory tests showed C-reactive protein of 37.8 mg/L (normal: less than 10 mg/L), white blood cell (WBC) of 11 × 10^9^/L (normal: 4.5-11 × 10^9^/L), neutrophils of 9.2 × 10^9^/L (normal: 2.5-7.5 × 10^9^/L), and hemoglobin of 13.4 g/dL (normal: 12.1-15.1 g/dL). The chest radiograph revealed that the esophageal stent was no longer located in the mediastinum (Figure 2).

**Figure 3:**
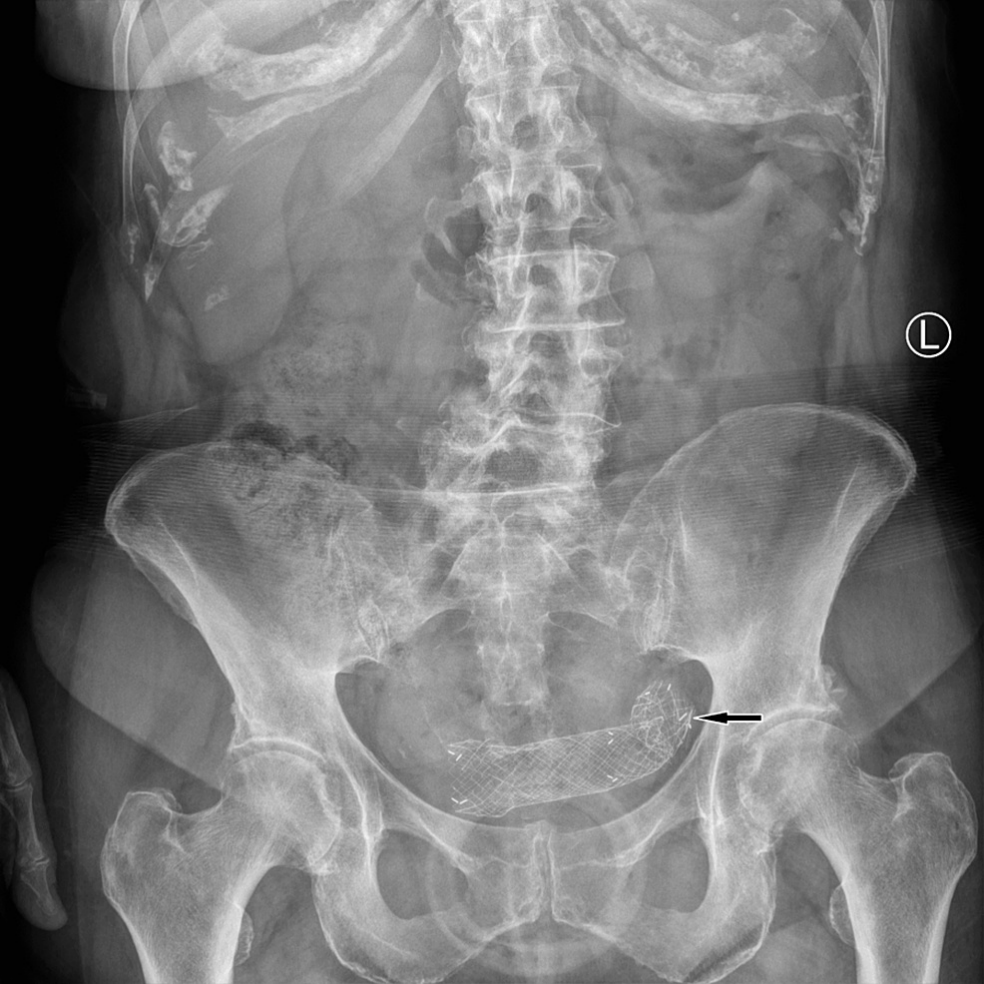
Plain film of the abdomen demonstrating the migrated stent (black arrow) located within the lower abdomen.

A plain film of the abdomen was performed to investigate the possible location of the stent and revealed its location superimposed over the pelvic inlet (Figure 3).

**Figure 4:**
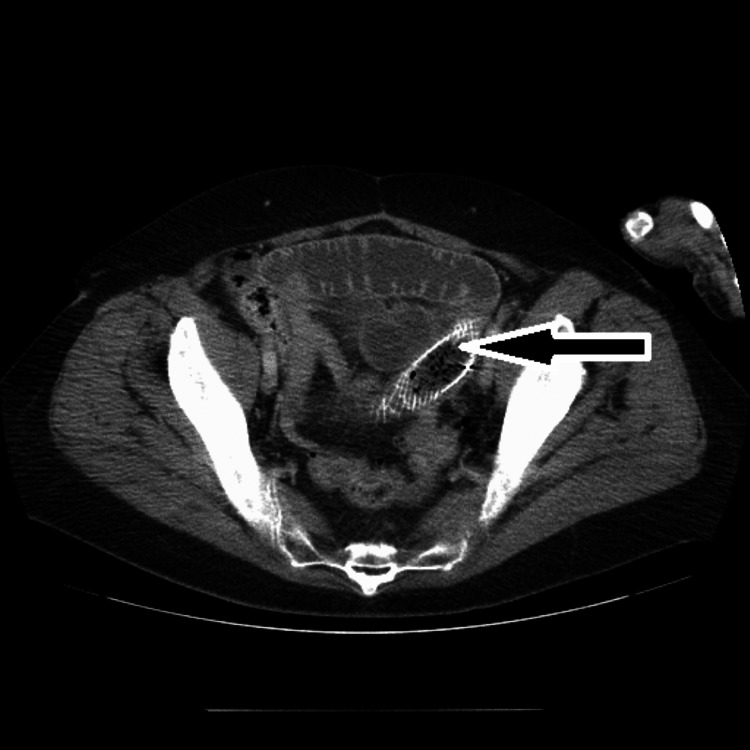
Axial CT demonstrating the stent (black arrow) located within the small bowel with proximal dilation. CT: computed tomography

**Figure 5:**
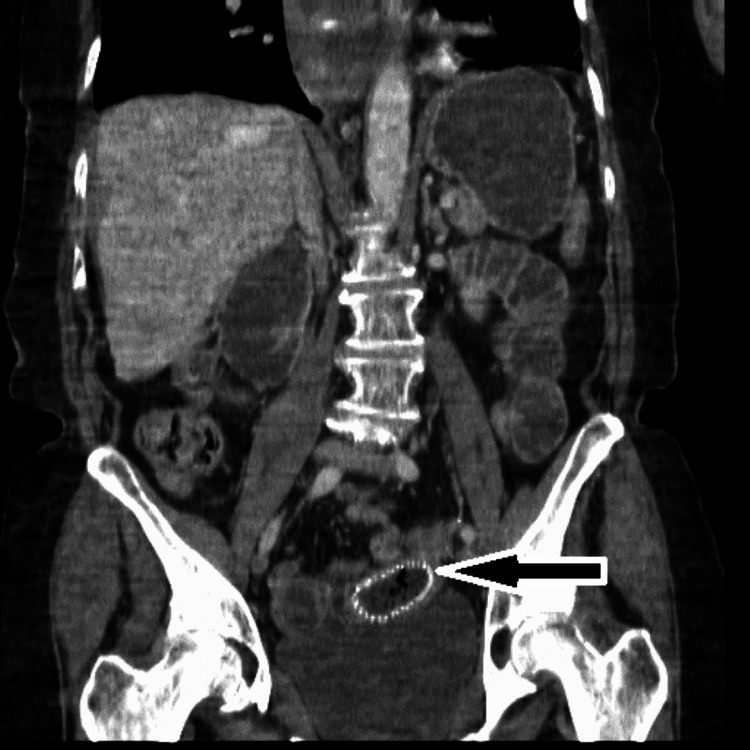
Coronal CT demonstrating the stent (black arrow) located within the small bowel. CT: computed tomography

Computed tomography (CT) showed that the stomach was distended with fluid, as well as the proximal small bowel. The bowel distention was demonstrated as far as the stent, which was located in the jejunum (Figure 4 and Figure 5).

She was managed with intravenous antimicrobials, nasogastric tube insertion, and intravenous fluid hydration. To assess if the stent may pass spontaneously, interval plain abdominal radiographs were performed on day 1 and day 3 post-admission.

With the lack of progression of the stent, she was planned for operative retrieval four days into admission. Laparoscopy was initially performed, but due to difficulty locating the stent, there was conversion to a midline laparotomy. Manual running of the small bowel located the stent within the distal jejunum.

**Figure 6:**
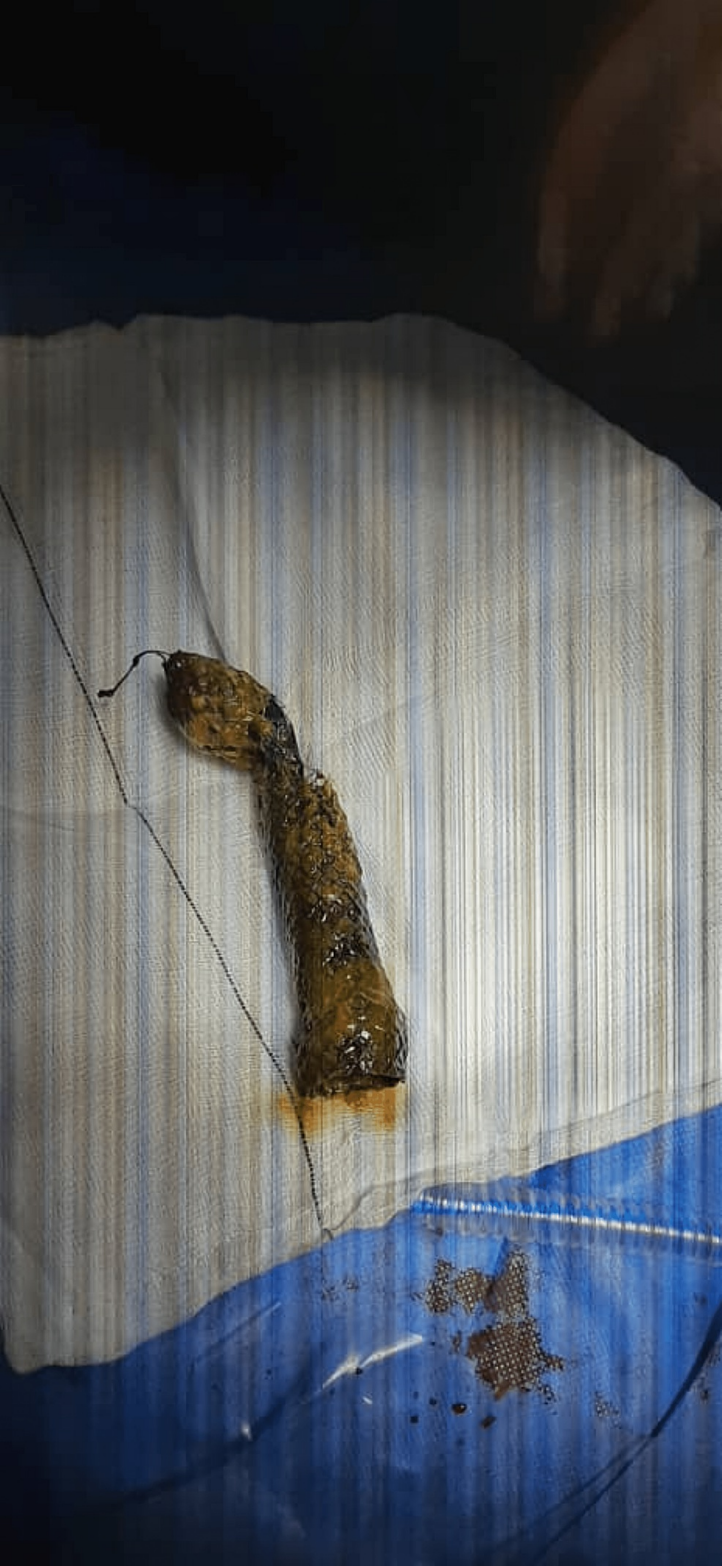
Image of the recovered stent with partial fracture.

Enterotomy was performed with retrieval of the stent, which was found to be incompletely fractured (Figure 6).

Resection of the involved small bowel was performed, followed by side-by-side stapler anastomosis using NTLC75 (Ethicon, Somerville, NJ, USA) and oversewn with 3-0 polydioxanone suture (PDS) (Ethicon, Somerville, NJ, USA). She was nil by mouth for 48 hours postoperatively with the nasogastric tube being removed at this time. Oral diet was initiated successfully three days postoperatively. She was well for discharge 10 days after surgery without any complications.

## Discussion

Esophageal stenting represents an important tool for the nonoperative management of esophageal pathologies including inoperable esophageal malignancies, benign strictures, and esophageal leakage [6,7]. Boerhaave’s syndrome is the spontaneous perforation of the distal esophagus due to high intraluminal pressures. Open surgical intervention has been the mainstay of management but is associated with a high burden of complications. Advances have been made with the use of therapeutic endoscopy with stent placement as discussed in this case [8]. The complications reported for esophageal stenting include pressure-induced ischemia, ulceration, perforation, development of new reactive stenosis bleeding or injury upon removal, and unsuccessful retrieval of the device at a later date [9]. Esophageal stent fracture is an uncommon complication, reported occurring anywhere from eight to 40 weeks after the initial stent placement [10]. The removal of the stent is generally recommended 4-6 weeks after initial placement [11,12]. The probability of stent migration varies from 3.7% to 50% depending on the diameter, type, coverage, and place of placement with up to 20% of covered stents migrating especially when placed at the esophageal gastric junction [13]. In the majority of cases, esophageal stents migrate no farther than the stomach where it is not associated with high morbidity [5]. The majority of migrated stents can be managed nonoperatively, being either removed endoscopically, exiting the body spontaneously, or remaining in the body in an uncomplicated state. Life-threatening complications of migration include hemorrhage, obstruction, perforation, and fistulation [13-15].

In this case, elective stent retrieval was attempted, and the stent was visualized on endoscopy but aborted due to difficulty in removing at endoscopy. The patient remained asymptomatic until 15 weeks after the trial of removal at endoscopy, where she presented here with small bowel obstruction secondary to the stent migrating and impacting the jejunum. Nonoperative management was attempted with serial abdominal radiograph imaging to assess for spontaneous passage of the stent, but with failure of spontaneous passage of the stent, operative surgery was performed. Stent migration requiring operative management is an uncommon occurrence with surgical methods involving laparotomy incision, enterotomy to retrieve the stent, local resection of the affected bowel, and primary anastomosis [16]. There are limited published cases with this, to the authors’ knowledge, being the first reported case of small bowel stent migration originally used for the nonoperative management of Boerhaave’s syndrome.

## Conclusions

Applications for endoscopic stenting remain an important nonoperative management option for both malignant and benign conditions of the esophagus. We present a case of small bowel obstruction secondary to esophageal stent migration, which was inserted for conservative management of Boerhaave’s syndrome. When possible, esophageal stents should be retrieved due to the complications associated locally and when migration occurs. Although surgical intervention is not commonly required, it is indicated when there is a risk of obstruction and perforation. We have demonstrated that this may be successfully managed with open retrieval of the stent, local bowel resection, and primary anastomosis.
